# Adolescents at Risk for Substance Use Disorders: Role of Psychological Dysregulation, Endophenotypes, and Environmental Influences

**Published:** 2008

**Authors:** Dawn L. Thatcher, Duncan B. Clark

**Keywords:** Adolescent, childhood, problematic alcohol use, alcohol use disorder (AUD), substance use disorder (SUD), heredity risk factors, environmental risk factors, behavioral phenotype, endophenotype, psychological dysregulation

## Abstract

Adolescents with alcohol-related problems often also use cigarettes and marijuana. Furthermore, early childhood characteristics that increase the risk for adolescent alcohol use disorders also increase the risk for problematic drug use. Identifying these characteristics early in childhood can be important for the prevention of alcohol and other drug (AOD) use disorders. As a result, researchers are seeking to identify liability factors and observable characteristics (i.e., phenotypes) that can predict substance use disorders (SUDs) across drug categories. Other studies are focusing on endophenotypes—characteristics that cannot be openly observed but which link a person’s genetic makeup, or genotype, and disease. Both predictive behavioral phenotypes and endophenotypes may reveal pathways connecting heritable predispositions and early environmental influences to later SUDs. One suggested predictive phenotype is psychological dysregulation, which is characterized by cognitive, behavioral, and emotional difficulties in childhood. An endophenotype that has been studied extensively is a particular brain wave called the P300 event-related potential. For people who are at high risk of AOD use based on these characteristics, adverse environmental conditions frequently lead to SUDs. Given the strong evidence that childhood psychological dysregulation predicts problematic AOD use, effective interventions for preventing adolescent SUDs may need to target the environmental features that put adolescents with this behavioral constellation at increased risk.

Adolescence is the developmental period of highest risk for the onset of problematic alcohol and other drug (AOD) use. Some experimentation with alcohol may be considered normal during adolescence; however, people who engage in binge drinking or who have developed alcohol use disorders typically also engage in other drug use, most frequently cigarettes and marijuana. AOD use behaviors are multifaceted and complex and are influenced by a multiplicity of genetic and environmental liabilities.

Risk factors for adolescent AOD use and substance use disorders (SUDs) can be conceptually divided into heritable, environmental, and phenotypic factors (Clark and Winters 2002). Heritable risk factors are reflected in familial patterns of SUDs and other psychiatric disorders. Environmental risk factors include family-related characteristics, such as family functioning, parenting practices, and child maltreatment, as well as other contextual factors, such as peer influences, substance availability, and consumption opportunities. These heritable and environmental factors then interact to determine a person’s observable characteristics and behaviors (i.e., phenotypes), such as AOD use. Therefore, understanding common genetic and environmental liabilities for adolescent AOD use is critical for developing effective prevention and intervention efforts. To better understand and to identify specific risk factors for adolescent SUDs, researchers have conducted longitudinal studies with high-risk children whom they followed through young adulthood.

One essential aspect of such studies is the choice of a suitable phenotype to study. A phenotype is an observable characteristic in a person that is the product of an interaction between the person’s genetic makeup (i.e., genotype) and environmental influences (Gottesman and Gould 2003). Most phenotypes are determined by multiple genes and environmental factors as well as by random variation. Accordingly, it can be difficult to select appropriate phenotypes that are relevant for a given genetic study. For example, the same genotype in several people might result in different phenotypes, depending on the environmental influences to which the people are exposed and random variation; likewise, the same phenotype might result from several different combinations of genes. Nevertheless, selection of an appropriate phenotype is a critical step in the process of characterizing adolescents who are at greatest risk for developing problematic alcohol and/or other drug use.

The phenotypes defined as adolescent SUDs can be understood as the culmination of developmental processes that are influenced by both shared and distinct genetic and environmental liabilities. The complexity and multiplicity of influences on SUDs and the problems that arise after the onset of problematic substance use make it difficult to identify the factors that lead to emergence of these phenotypes. Because an understanding of these factors is critical to the development of more effective prevention and intervention programs, researchers have started to look for phenotypes that arise early in childhood and which may provide insight into the causes of SUDs. One advantage of looking for phenotypes that develop early in life is that predictive childhood behavioral phenotypes may be more closely related to underlying genetic factors (i.e., genotypic liabilities) than predictive adolescent phenotypes, simply because environmental influences have had less time to shape the behavior of children compared to adolescents.

Recent studies (e.g., Tarter et al. 1999, 2003) have identified a construct referred to as “childhood psychological dysregulation” as a behavioral phenotype that reflects a person’s general liability of developing AOD problems in adolescence. (This phenotype will be described in more detail in the following section.) For people with this liability, adverse environmental characteristics often lead to the development of SUDs. Furthermore, researchers have identified other, covert characteristics known as endophenotypes^1^ that link a specific genotype with a behavioral phenotype or disease. For example, specific neurobiological endophenotypes, such as a type of brain wave known as the P300 event-related potential (ERP), may constitute the underpinnings of psychological dysregulation.

This article reviews risk factors for adolescent SUDs in the context of a conceptual model that considers predictive behavioral phenotypes such as psychological dysregulation and endophenotypes, like the P300 response, to be of central importance. The article also discusses the role of various environmental risk factors and of the aggregation of risk factors. Finally, the implications of these findings for the development of prevention and intervention approaches are addressed.

## Heritable Risks

Historically, a person’s genetic risk for developing a certain disorder has been estimated by establishing a family history of the disorder, and this approach remains important for research on SUDs. Presence of an SUD in a parent has consistently been shown to be a strong risk factor for adolescent AOD use and SUDs. However, the transmission of SUDs from parent to offspring occurs through both genetic and environmental influences (Sartor et al. 2006).

In general, children of alcoholic parents (COAs) have been studied more extensively than children of parents with other addictive disorders. The existing studies identified both common and distinct features between COAs and children of parents with other SUDs. For example, compared with children whose parents have no SUDs (i.e., reference children), COAs exhibit increased rates of alcohol use disorders (Schuckit and Smith 1996). Similarly, children of parents with SUDs involving cocaine, heroin, or other illicit drugs tend to start using tobacco earlier than reference children and to have increased rates of illicit drug use and SUD symptoms (Clark et al. 1999).

Although it is important to identify risk factors for specific SUDs, there has been an increasing emphasis on finding common liabilities for all SUDs because researchers hope that this focus will help identify new targets for prevention efforts. As mentioned earlier, childhood behavioral phenotypes predictive of later AOD use and SUDs are more closely related to genetic liability and effects of parental AOD use than are behavioral phenotypes that emerge later in life. One childhood behavioral phenotype that has provided a conceptual structure for developing common liability models is the construct of psychological dysregulation. As reviewed in the following sections, the phenotype of psychological dysregulation is related to parental characteristics, is predictive of adolescent outcomes, and may be a manifestation of neurobiological endophenotypes.

## Psychological Dysregulation

Psychological dysregulation is defined as deficiency in three domains—cognitive, behavioral, and emotional—when adapting to environmental challenges. These three domains of dysregulation are statistically related to one overall dimension, conceptualized as psychological dysregulation (Tarter et al. 2003). Variations in psychological dysregulation at specific developmental stages may be important for understanding adolescent SUDs (Clark and Winters 2002). For example, an increasing number of studies indicate that childhood psychological dysregulation predicts adolescent SUDs (e.g., Tarter et al. 1999). Furthermore, childhood psychological dysregulation significantly discriminates boys with and without parental SUDs (Tarter et al. 2003).

As described by Tarter and colleagues (1999, 2003), psychological dysregulation can be thought of as a single construct that comprises distinct but related components—executive cognitive dysfunction, behavioral impulsivity, and emotional lability. However, it also is important to note that alternative conceptualizations of these dimensions and their relationship to childhood and adolescent disorders exist. For example, Nigg (2000) presented a taxonomic model of different categories of inhibition and disinhibition based on cognitive and personality factors and reviewed how different components of these factors relate to various disorders and hypothesized brain structure and function. This work provides a useful context for the following presentation of psychological dysregulation, which includes factors of cognitive disinhibition as well as incorporating emotional and behavioral components.

The view that psychological dysregulation is one construct comprising several components (i.e., a unitary concept) also is consistent in several important ways with another concept—the externalizing spectrum—which was proposed by Krueger and colleagues (2002). Using data from studies of twins as well as mother reports, these investigators found evidence for a hierarchical model linking SUDs, conduct disorder (CD), and antisocial personality disorder. In a factor analysis, these disorders all were related to one general factor, which the investigators labeled an “externalizing factor.” In addition to this general factor, the model also posits that distinct etiological characteristics pertaining to each disorder are involved. This is a critical point because although the single phenotypic factor of psychological dysregulation is thought to be related to adolescent SUDs and many other disorders, distinct etiological factors (which may not even have been elucidated yet) likely also influence the specific outcome during adolescence.

In its more severe forms, psychological dysregulation during childhood manifests as disruptive behavior disorders, such as CD, oppositional defiant disorder (ODD), and attention deficit/hyperactivity disorder (ADHD). However, psychological dysregulation in different manifestations can be observed at all developmental stages, including CD and ADHD during childhood, SUDs during adolescence, and borderline personality disorder or antisocial personality disorder during adulthood.

CD during childhood is one of the most important predictors of adolescent SUDs (Bukstein 2000; Clark et al. 2002; Sartor et al. 2006). Among a sample of about 500 boys, 250 of whom demonstrated antisocial behavior, White and colleagues (2001) investigated associations among early psychopathology and trajectories of AOD use during adolescence. The investigators found that higher levels of several disorders—including CD, ODD, ADHD, and depression—predicted higher levels of alcohol use, although only CD predicted increased alcohol use over time. In a different study of 177 adolescent boys with and 203 boys without paternal SUDs, Clark and colleagues (1999) demonstrated that antisocial disorders, including CD and ODD, partially were responsible for the relationship between paternal SUDs and substance-related problems during adolescence.

However, it is not only psychological dysregulation found in a child that may predict SUDs when that child reaches adolescence.

Psychological dysregulation in that child’s parents during their childhood also may contribute to the heritable risks for SUDs (Clark et al. 2004*a*).

The cognitive dimension of psychological dysregulation, also known as executive cognitive dysfunction, is particularly relevant for understanding SUDs (Giancola and Tarter 1999). For example, in a study of 66 high-risk adolescents, Tapert and colleagues (2002) demonstrated that a high level of executive cognitive dysfunction^2^ predicted AOD use and SUDs 8 years later, even when controlling for other factors, such as level of baseline AOD use, family history of SUDs, and CD in the child. Executive cognitive function might be one of the primary components underlying the relationship between psychological dysregulation and AOD involvement.

In summary, the construct of psychological dysregulation strongly predicts AOD use initiation, acceleration, and related problems during adolescence. As described in the following section, various brain structures, such as the prefrontal cortex in the outer layer of the brain and subcortical regions located deeper within the brain, may be involved in the development of psychological dysregulation. In other words, these brain structures may be neural substrates of psychological dysregulation.

## Brain Structures Related to Psychological Dysregulation as Endophenotypes

Chambers and colleagues (2003) have described adolescence as the “critical period of addiction vulnerability” (p. 1042), because during this period the brain pathways (i.e., neural circuits) that enable people to experience motivation and rewarding experiences still are developing. These pathways include, among others, regions called the anterior prefrontal cortex and ventral striatum (see figure).^3^ Moreover, the adolescent and adult brains appear to differ with respect to the brain regions that primarily respond to novel stimuli. It appears that the adolescent brain responds to novel stimuli largely through a brain structure known as amygdala. This is part of the brain’s limbic system, which, among other functions, is involved in controlling emotions. In contrast, the adult brain increasingly uses higher cognitive functions (i.e., executive functions) mediated by the frontal cortex to interpret novel stimuli (Ernst and Paulus 2005). Variations in how these neural pathways develop may contribute to the risk for AOD drug use during adolescence. Specifically, researchers have suggested that psychological dysregulation may be related to the function of the prefrontal cortex (Dawes et al. 2000).

As mentioned earlier, endophenotypes are characteristics that cannot be observed with the naked eye but can be measured using other techniques and which are part of the pathway from a person’s genotype to an observable behavioral phenotype or outcome (Gottesman and Gould 2003). A well-accepted endophenotype in psychiatry is a certain brain wave called the P300 ERP, mentioned earlier. ERPs are a series of changes in normal electrical brain activity that occur after a person is exposed to a sudden stimulus, such as a sound or light, and are a measure of brain activity during the processing of new information. They can be recorded using an electroencephalograph (EEG). One of the most consistent components of the ERP occurs approximately 300 milliseconds after a novel and rare stimulus and is therefore called P300 (Bauer and Hesselbrock 1999). It is one of the most commonly used ERP components in the study of the effects of AODs on cognitive functions.

Studies found that in adolescent boys a reduced P300 amplitude^4^ is associated with disorders reflecting psychological dysregulation in the father and predicts the development of SUDs by young adulthood (Iacono et al. 2002).^5^ The relationship between low P300 amplitudes during childhood and increased risk of SUDs in young adulthood appears to be mediated by behavioral problems during childhood and adolescence—that is, adolescents with lower P300 amplitudes also had more behavioral problems during childhood and adolescence and also were more likely to develop SUDs during young adulthood (Habeych et al. 2005).

A large study of identical and fraternal twins—the Minnesota Twin Family Study—has provided substantial support for the notion that the P300 response can serve as an endophenotype for adolescent SUDs. For example, the study investigators found that the P300 response was strongly heritable and showed strong relationships to many other phenotypic predictors of adolescent SUDs, such as early or frequent cannabis use (Yoon et al. 2006).

Although less intensively studied, other brain structures and functions also may represent endophenotypes that can be helpful in understanding the link between genes, the environment, and adolescent SUDs (Glahn et al. 2007). One of these proposed endophenotypes is the development of white matter in the brain, which consists of nerve cell extensions (i.e., axons) that connect neurons to other neurons located in the same or other brain regions. This potential endophenotype still needs to be investigated further.

## Environmental Influences on Risk of Adolescent SUDs

Several environmental influences have been identified that affect the risk of accelerated AOD involvement and the development of adolescent SUDs. As described in the following sections, major environmental influences include child maltreatment and other traumatic events; parental influences, such as parenting practices; and peer influences. Some of these also lead to manifestations of psychological dysregulation, such as CD, ADHD, and major depressive disorder.

Moreover, it is important to note that the risk factors that contribute to the initiation of AOD use likely are distinct from those factors that contribute to the progression from initial use to regular use and ultimately to diagnosed SUDs (Clark and Winters 2002; Donovan 2004). This also applies to the relative importance of genetic versus environmental risk factors. A recent population-based twin study (Fowler et al. 2007) demonstrated that environmental variations (such as the ones described below) were relatively more influential for the timing of the initiation of substance use, whereas genetic variations (such as the ones described earlier) were more influential in accelerating the progression from initiation of use to heavier use. In any case, early initiation of AOD use has been well established as a risk factor for adolescent SUDs (e.g., Clark et al. 2005*a*).

### Maltreatment and Other Traumatic Events

Child maltreatment and the experience of other traumatic events provide an environmental context that modifies the genetically determined phenotypic predictors of adolescent SUDs, such as psychological dysregulation. Moreover, these experiences also may influence the emergence of the phenotypic behaviors by inducing psychophysiological changes in the hormone system that mediates the body’s response to stress, known as the hypothalamic–pituitary–adrenal (HPA) axis. (For more information on the HPA axis, the stress response, and its relationship to AOD use and SUDs, see the article by Wand, pp. 119–136.)

The finding that child maltreatment has an impact on the development of adolescent SUDs has been supported by several studies. For example, in a study by Clark and colleagues (1997), adolescents with SUDs retrospectively reported higher incidences of childhood maltreatment, including physical and/or sexual abuse, than adolescents without SUDs. In another study involving more than 3,500 female twins, Sartor and colleagues (2007) found childhood sexual abuse to be associated with higher rates of alcohol use and dependence. Finally, in a longitudinal study of 76 maltreated children and 51 demographically matched control children, Kaufman and colleagues (2007) determined that maltreated children were seven times more likely to report alcohol use at age 12 than were the control children; moreover, maltreated children used alcohol an average of 2 years earlier than did the control children. The investigators of that study also collected genetic information on the children and observed a gene-by-environment interaction, whereby the presence of a particular variant of one gene^6^ in children who were maltreated was associated with the highest risk for alcohol use.

Although child maltreatment is clearly associated with adolescent SUDs, the mechanisms underlying this relationship are less clear. They likely involve both psychological and physiological responses. From a psychological perspective, traumatic events such as child maltreatment may lead directly to AOD use because the affected person attempts to self-medicate the anxiety and depression resulting from the traumatic event. Additionally, the effect of child maltreatment is related to and may be confounded by parental SUDs—that is, parents with SUDs may be more likely to mistreat their children and also are likely to pass on a genetic predisposition to AOD use.

### Parenting Practices

Low levels of parental monitoring are a significant predictor of adolescent SUDs. A study based on the Monitoring the Future data from 1994 to 1996 (Johnston et al. 2004) found that parental involvement significantly predicted AOD use in the past 30 days across all age, gender, and ethnic groups (Pilgrim et al. 2006). The association of parental monitoring and both alcohol and marijuana use also has been demonstrated in a sample of low-income teens in a health clinic setting (DiClemente et al. 2001). Moreover, this relationship is found regardless of whether parental monitoring is assessed based on adolescents’ perceptions or on adult reports of monitoring (Griffin et al. 2000). Finally, in a prospective study, Clark and colleagues (2004*a*, 2005*b*) found that among community adolescents who had never had an SUD, those who reported low levels of parental supervision were more likely to subsequently develop an alcohol use disorder.

The relationship between parenting practices and adolescent SUDs may result, in part, from the effects of parental SUDs, which contributes to such environmental influences as inadequate parental involvement and modeling of substance use. For example, Barnes and colleagues (2000) demonstrated that the effects of parental alcohol abuse on adolescent alcohol use were largely through inadequate monitoring and inadequate emotional support behaviors. In general, three types of factors have been found to contribute to the increased risk of AOD use observed among COAs: genetic factors such as the ones described earlier in this article, alcohol-specific parenting factors, and non–alcohol-specific parenting factors (Ellis et al. 1997; Jacob and Johnson 1997). Alcohol-specific parenting factors include direct modeling of drinking and drug use behavior as well as shaping of alcohol expectancies.^7^ Non–alcohol-specific factors placing children at greater risk of AOD use include coexisting other psychological disorders or cognitive dysfunction in the parents, low socioeconomic status, and increased family aggression and violence.

### Peer Influences

Peers are an important environmental factor in the development of adolescent SUDs, although peers seem to have a more modest role relative to parents. Longitudinal studies have demonstrated that peer AOD use predicts adolescent alcohol use (Bray et al. 2003) and marijuana use (Brook et al. 1999). Moreover, affiliation with peers who generally engage in deviant behaviors predicted adolescent SUDs in a longitudinal study (Cornelius et al. 2007). Other studies suggested that affiliation with deviant peers might mediate relationships between early risk factors (e.g., temperament) and subsequent AOD use and SUDs (Giancola and Parker 2001). One issue plaguing research in this area is the difficulty in determining whether peer effects result from modeling—that is, that the adolescent copies the behavior of his or her peers, which would be more of a true environmental influence—or from selection—that is, that adolescents who already are predisposed to accelerated substance involvement because of other factors naturally seek out like-minded peers. A longitudinal study of more than 6,000 adolescents found that peer alcohol use at the beginning of the study was significantly related to increases in adolescent alcohol use over time; moreover, the reverse also was true in that adolescent alcohol use at the beginning of the study also was related to increases in peer alcohol use (Bray et al. 2003). This study suggests that both directions of influence likely contribute to the association of adolescent and peer AOD use.

## Aggregation of Risk Factors

Risk studies typically have assessed the impact of one or more variables on risk in a large group of people, often at only one point in time. Conversely, longitudinal studies that follow individual people over time have typically focused on single risk factors, such as parental AUDs. Relatively few studies, however, have examined multiple risk factors over time in a way that is directly applicable to specific individuals.

A few informative studies have demonstrated how multiple factors from different domains combine to determine an individual’s risk for accelerated AOD use and SUDs. For example, Clark and colleagues (2005*b*) used an approach called cluster analysis to divide 560 children into five risk groups. For this analysis, the investigators used the variables of parental SUDs early use of alcohol or tobacco; and psychological dysregulation integrated across affective, behavioral, and cognitive domains. The analysis found that the lowest-risk group was defined by having no parent with an SUD, no early substance use, and low levels of psychological dysregulation. At the other extreme, the highest-risk group was defined by having two parents with an SUD, early use of one or two substances, and the highest levels of psychological dysregulation. The children in this highest risk group demonstrated significantly earlier use of and associated problems with tobacco, alcohol, marijuana, and cocaine. These results demonstrate that it may be necessary to combine multiple risk characteristics to comprehensively identify the children and adolescents at greatest risk of accelerated AOD use.

## Prevention and Intervention

The available research shows that childhood manifestations of psychological dysregulation, including affective dysregulation and irritability, behavioral impulsivity and CD, and executive cognitive dysfunction, predict problematic AOD use during adolescence. These results have important implications for the design of prevention and intervention programs to be delivered during childhood and adolescence. Given the limited funds available for prevention programs, targeting children who exhibit early indicators of psychological dysregulation seems a reasonable starting point. Research has demonstrated that children with conduct problems or CD, early cigarette or other substance use, and parents who have SUDs are extremely likely to engage in problematic AOD use by early adolescence. These three criteria are relatively straightforward to assess on an individual basis in schools or in primary care settings.

Briones and colleagues (2006) summarized the biopsychosocial model of adolescent SUDs and made specific recommendations for prevention efforts. From a biological perspective, the investigators recommended that detection and treatment of early symptoms of CD and ADHD should be accomplished as early as possible by a primary care provider and/or child psychiatrist. Additionally, education of children and adolescents, their families, schools, and broader communities about the increased risk to children of parents with SUDs is critical for effective prevention. Notably, Kelley and Fals-Stewart (2008) found secondary effects of treatment of parental SUDs in terms of diminished externalizing behavior in their young children but not in adolescent children, supporting the notion that intervention is particularly effective in families with young children.

From a psychological perspective, Briones and colleagues (2006) recommended early and frequent screening for problematic alcohol use during late childhood and early adolescence using established screening instruments and established cutoff scores (see, for example, Chung et al. 2000). Finally, from a social perspective, Briones and colleagues (2006) noted that adolescents who engage in positive social activities such as organized sports, volunteer activities, and religious activities are less likely to engage in AOD use and develop SUDs than adolescents who do not engage in such activities. However, these studies are cross-sectional in nature—that is, they assess a large group of adolescents at one time and do not follow specific adolescents over time to see which factors affect their development. Moreover, the causes underlying this observed relationship still are unclear.

Several observations also suggest that prevention programs should be as comprehensive as possible. For example, researchers have discovered that liability for AOD-related problems is not linked to one particular substance but that many aspects of liability are common to all forms of AODs. Therefore, focusing programs on one particular drug likely is not an effective prevention method. However, given the escalation from cigarette use to AOD involvement and dependence among high-risk adolescents, treatment programs that discourage the use of cigarettes might well enhance outcomes. Furthermore, prevention programs that have demonstrated some success (e.g., Mason et al. 2003) often include multiple levels of intervention, including the adolescents, parents, families, and communities.

As discussed in this article, parental influences—particularly low levels of parental monitoring—are strongly associated with accelerated substance involvement among adolescents. These associations exist regardless of the SUD status of the parents, and parenting behaviors seem to be a significant environmental mechanism mediating the association between parental alcohol use disorders and adolescent alcohol involvement and problems. Accordingly, parental involvement has been shown to be critical to the success of adolescent alcohol prevention and treatment programs. Specifically, high levels of parental supervision and communication are associated with better outcomes after treatment (Thatcher and Clark 2004).

With respect to the timing and targeting of intervention efforts, it is important to note that not all adolescents who engage in AOD use necessarily require treatment. In fact, modest AOD use has been considered to be a typical event during adolescence and does not necessarily presage the development of SUDs (Kaminer 1999). Instead, it is essential to consider risk factors identifiable much earlier in the developmental process in conjunction with the timing and nature of adolescent AOD use.

By the time adolescents do require treatment for SUDs, they are well on their way through the developmental stages during which risk emerges. At that point, it is helpful to know the various risk factors that resulted in the adolescent’s heavy substance use. In any case, the first step of treatment is to engage the adolescent in the treatment process. This often is challenging, given that adolescents frequently experience external pressures to enter treatment and are not themselves motivated to change their problematic behavior (Battjes et al. 2003). However, adolescents’ motivation to engage in treatment can be enhanced by increasing their awareness of the negative consequences of substance use (Battjes et al. 2003).

Because adolescents with SUDs typically have multiple problems, treatment programs focusing primarily on abstinence from AODs may be insufficient. Rather, multimodal programs addressing various aspects of psychological dysregulation—including behavior modification, management of affect, and perhaps cognitive rehabilitation—are optimal. Multimodal programs, including “multisystemic therapy” as presented by Henggeler and colleagues (1999), have been shown to be effective in adolescents exhibiting severe SUDs in addition to antisocial behavior. Furthermore, practice guidelines for the treatment of adolescent alcohol use disorders (Bukstein 1998) emphasize that effective treatments must be of an intensity and duration that produces both external (i.e., behavioral) and internal (i.e., emotional and attitudinal) change.

In summary, effective treatment programs for adolescent SUDs are likely to be multimodal, to involve the family to the extent possible, and to focus on aspects of AOD involvement as well as related problems.

## Figures and Tables

**Figure:**
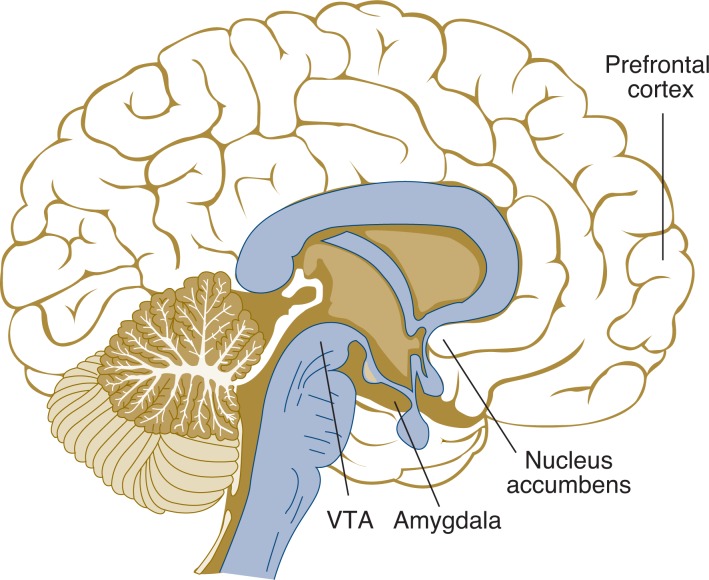
Location of the brain regions related to psychological dysregulation. One neural circuit, which is involved in motivation and mediating rewarding experiences, includes the ventral tegmental area (VTA), the nucleus accumbens (which is part of a larger structure called the ventral striatum) and the prefrontal cortex. Another group of structures that is involved in the response to novel stimuli is the amygdala.

## Footnotes

1 An endophenotype is defined as an “internal phenotype”—that is, a characteristic that is not obvious to the naked eye but which can be measured through, for example, biochemical tests or microscopic examination (see Gottesman and Gould 2003). Thus, endophenotypes can be neurophysiological, neuroanatomical, biochemical, etc., in nature. These endophenotypes provide a link between a person’s genotype and more complex overt phenotypes, such as a disease with numerous symptoms. Investigation of endophenotypes allows researchers to study the genetic underpinnings of a complex disease such as AOD dependence by focusing on simpler facets or traits associated with the disease.

2 Executive cognitive functions are brain processes — such as planning, abstract thinking, or inhibiting inappropriate behavior—that guide a person’s conscious thoughts and behavior. Cognitive dysfunction in this study was characterized by low attention and low scores on executive cognitive function.

3 This pathway also is known as the dopaminergic meso-corticolimbic pathway. For more information on this pathway and its functions, see the article by Wand, pp. 119–136.

4 The term “amplitude” refers to the height of the P300 wave.

5 Maternal substance use during pregnancy does not appear to contribute to this relationship.

6 The variant involved was the short allele (s-allele) of the serotonin transporter (HTTLPR) gene.

7 Alcohol expectancies are the effects that a person expects alcohol to have (e.g., to relieve anxiety or to lead to uncontrolled behavior).
